# No significant impact of patient age and prior treatment profile with docetaxel on the efficacy of cabazitaxel in patient with castration-resistant prostate cancer

**DOI:** 10.1007/s00280-018-3698-1

**Published:** 2018-10-03

**Authors:** Takeo Kosaka, Hiroshi Hongo, Keitaro Watanabe, Ryuichi Mizuno, Eiji Kikuchi, Mototsugu Oya

**Affiliations:** Department of Urology, Keio University School of Medicine, 35 Shinanomachi, Shinjuku-ku, Tokyo, 160-8582 Japan

**Keywords:** Castration-resistant prostate cancer, Chemotherapy, Cabazitaxel, Age, Docetaxel

## Abstract

**Background:**

The correlation of the oncological outcomes of docetaxel and cabazitaxel in Japanese metastatic castration-resistant prostate cancer (mCRPC) patients has not been unclear.

**Materials and methods:**

This study included a total of 47 consecutive Japanese mCRPC patients treated with cabazitaxel and assessed the prognostic significance of cabazitaxel, focusing on patient age and the correlation of efficacy between docetaxel and cabazitaxel.

**Results:**

Prostate-specific antigen (PSA) decline was observed in 27 patients (57.4%), including 19 (40.0%) achieving the response defined by PSA decline ≥ 30%. The median overall survival (OS) periods after the introduction of cabazitaxel was 16.1 months. Twenty (42.6%) were judged to have responded to cabazitaxel with a PSA decrease ≥ 30% from the baseline. A 30% PSA response to cabazitaxel was achieved in 4 (50.0%) patients with ≧ 75 years (*n* = 8) and 16 (41.0%) patients with less than 75 years (*n* = 39). There was no significant correlation between the PSA response and patients’ age (*p* = 0.707). A 30% PSA response to cabazitaxel was achieved in 13 (46.4%) and 7 (36.8%) patients with and without that to docetaxel, respectively. A 30% PSA response to cabazitaxel was achieved in 5 (16.6%) and 7 (41.2%) patients who had treated with less than 10 cycles docetaxel or 10 ≦ cycles, respectively. Univariate and multivariate analyses revealed that there were no significant correlation of patient age (*p* = 0.537), the response to prior docetaxel therapy (*p* = 0.339) or cycles of docetaxel therapy (*p* = 0.379) with shorter OS.

**Conclusion:**

These results indicate that the introduction of cabazitaxel for Japanese mCRPC patients could result in oncological outcomes without any association with patient’s age and the profiles of previous docetaxel therapy.

**Electronic supplementary material:**

The online version of this article (10.1007/s00280-018-3698-1) contains supplementary material, which is available to authorized users.

## Introduction

Cabazitaxel is a next-generation taxane which indicated for the treatment of patients with metastatic castration-resistant prostate cancer (mCRPC) previously treated with a docetaxel-containing regimen [1–3]. Phase III TROPIC study that enrolled patients up to the age of 80 years revealed cabazitaxel provided an overall survival (OS) benefit in patients with mCRPC progressing after docetaxel. Cabazitaxel was approved worldwide for the treatment of patients with mCRPC previously treated with a docetaxel-containing regimen [4–7]. In a phase I study of cabazitaxel in patients with mCRPC in Japan that enrolled patients up to the age of 74 years, PK parameters, safety and tolerability in Japanese patients were found to be comparable to results of previous studies in Caucasian patients, and the MTD was identified as 25 mg/m^2^ [8, 9]. However the safety and antitumor activity in higher-age patients more than 74 years have not been fully characterized yet. Moreover, the correlation of the oncological outcomes of docetaxel and cabazitaxel in Japanese mCRPC patients has not been unclear.

The aim of the present study was to investigate the oncological outcomes of the patients treated with cabazitaxel in Japanese mCRPC patients, focusing on patient’s age and prior treatment profile with docetaxel.

## Materials and methods

In this retrospective observational study, 47 patients with mCRPC treated with cabazitaxel at Keio University Hospital from 2014 to 2017 were included. All patients were histologically confirmed as having adenocarcinoma of the prostate with radiologic evidence of metastatic disease and had disease progression during treatment consisting of complete androgen blockade hormone therapy and docetaxel. All patients received cabazitaxel at 20–25 mg/m^2^ administered intravenously on day 1 of each treatment cycle, together with prednisone 5 mg twice daily. Prophylactic administration of G-CSF was prescribed to all the patients.

For the objective of this study, prior treatment profile with docetaxel and laboratory data of each patients prior treatment profile were retrospectively obtained from the medical records. Docetaxel was generally given at a dose of 75 mg/m^2^ every 3 weeks. Dose modification or treatment delay was permitted based on treatment-associated adverse events (AEs). A 30% PSA response to docetaxel was defined as a response to docetaxel in mCRPC patients. PFS was defined as an increase in PSA values ≥ 25% relative to the pretreatment PSA value or radiological progression according to the RECIST guidelines. Overall survival (OS) was calculated from the date of start of cabazitaxel treatment to the date of death or date of last follow-up. Adverse events (AE) were classified according to CTCAE dictionary version 4.0.

Our study was designed as a retrospective analysis and approval was obtained from the Institutional Review Board of our institution.

### Statistical analysis

The continuous variables and categorical variables of different groups were compared using the Chi square test and Mann–Whitney *U* test, respectively. The Kaplan–Meier method was used to estimate the event-time distributions for PFS and OS, and the log-rank test was then used to assess the significance. Univariate Cox regression models were used to adjust for potential confounders in predicting OS. Categorized variables were assessed in multivariate models using Cox proportional hazard regression models with a stepwise forward selection method. For all statistical analyses, tests were two-sided and *p* < 0.05 was considered to indicate statistical significance. All statistical analyses were performed using the Statistical Package of the Social Sciences, version 24.0 (SPSS, Chicago, IL, USA).

## Results

### Patient characteristics

**Fig. 1:**
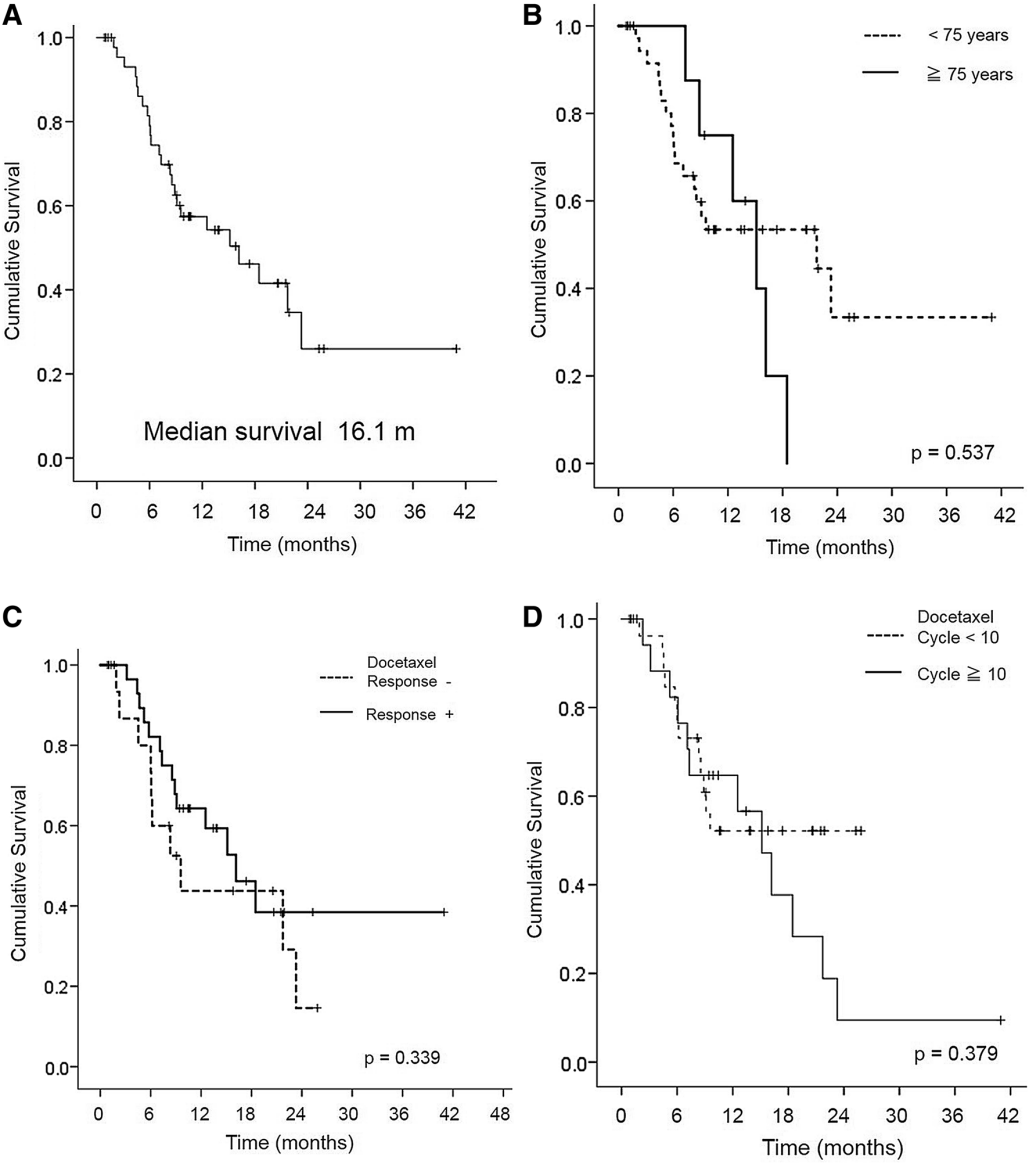
**A** Kaplan–Meier for time-to-overall survival in total population (*n* = 47). **B** Kaplan–Meier for time-to-overall survival in the patient age-specified population (*n* = 47). **C** Kaplan–Meier for time-to-overall survival in the docetaxel cycle number-specified population (*n* = 47). **D** Kaplan–Meier for time-to-overall survival in the docetaxel response-specified population (*n* = 47)

The summary of 47 mCRPC patient characteristics is shown in Table 1. Median age was 71 years. The ECOG PS score was 0 and 1/2 in 83.0% and 17.0% of patients, respectively. The median baseline PSA level was 124.3 ng/mL (range 0.17–11660). Major sites of disease included bone (97.8%). The median prior docetaxel cycle was 8 (range 3–43). Cabazitaxel was applied as the second- or third-line treatment in 11 (23.4%) patients and as fourth-line or more in 37 (78.7%) patients. Treatment was generally well tolerated with a median of 5 cycles (range 1–30) (Table 2). During the observation period of this study (median 16.2 months; range 2–44 months), the median OS periods of the 47 patients was 16.1 months (Fig. 1A).

**Table 1:** Characteristics of patients treated with cabazitaxel

	*N* = 47
Age, years, median (range)	70 (46–85)
Age group, *n* (%)	
< 75 years	39 (83.0)
75-years	8 (17.0)
ECOG PS, *n* (%)	
0	40 (84.4)
1, 2	7 (15.6)
PSA at baseline, ng/mL, median (range)	124.3 (0.17–11660)
Metastatic sites involved, *n* (%)	
Bone	46 (97.9)
Lymph nodes	18 (38.3)
Visceral metastasis	13 (27.7)
EOD score	
< 2	28 (59.6)
2 ≦	26 (55.3)
Prior surgery, *n* (%)	10 (21.3)
Prior radiotherapy, *n* (%)	20 (42.6)
Prior second AR targeting lines, *n* (%)	
ENZA/ABI	29 (61.7)/22 (46.8)
Prior docetaxel-containing regimens, *n* (%)	
1, 2	11 (23.4)
3	17 (36.2)
4 or more	17 (36.2)
Total prior docetaxel cycle, median (range)	8 (3–43)
PSA at baseline, ng/mL, median (range)	124.3 (0.17–11660)
Hb at baseline, g/dl, median (range)	11.5 (9.0-14.8)
ALP at baseline, IU/L, median (range)	276 (113–3146)

*ECOG PS* Eastern Cooperative Oncology Group performance status, *ENZA* Enzalutamide, *ABI* abiraterone, *Hb* hemoglobin

**Table 2:** **(**a) Treatment-emergent grade 3/4 adverse events (TEAEs) of patients and (b) laboratory abnormalities in patients treated with cabazitaxel (*N* = 47)

Preferred term	*n* (%)
	Grade 3/4
(a) Non-hematologic laboratory abnormalities	
Fatigue	7 (14.9)
Anorexia	4 (7.3)
Nausea	3 (6.4)
(b) Hematologic laboratory abnormalities	
Neutrophil count decreased	24 (51.1)
Anemia	6 (12.8)
Platelet count decreased	3 (6.4)
Febrile neutropenia	3 (6.4)

Following treatment with cabazitaxel therapy, and 20 (42.6%) were judged to have responded to cabazitaxel with a PSA decrease ≥ 30% from the baseline. A 30% PSA response to cabazitaxel was achieved in 4 (50.0%) patients with ≧ 75 years (*n* = 8) and 16 (41.0%) patients with less than 75 years (*n* = 39). There was no significant correlation between the PSA response and patients’ age (*p* = 0.707). A 30% PSA response to cabazitaxel was achieved in 13 (46.4%) and 7 (36.8%) patients with and without that to docetaxel, respectively. There was no significant correlation of the PSA response between docetaxel and cabazitaxel (*p* = 0.561). A 30% PSA response to cabazitaxel was achieved in 5 (16.6%) and 7 (41.2%) patients who had been treated with less than 10 cycles docetaxel or 10  ≦ cycles, respectively. There was no significant correlation of the PSA response between docetaxel and cabazitaxel (*p* = 0.226).

### Univariate and multivariate analysis of OS

Our primary objective was to examine whether the patient age or prior treatment profile with docetaxel had any associations between OS in men with mCRPC receiving cabazitaxel. To identify the clinical–biological parameters associated with OS in patients treated with cabazitaxel chemotherapy, univariate and multivariate analyses were performed using a Cox proportional hazard regression model.

Univariate analysis revealed that poor PS (*p* < 0.001), Hb < 11 mg/dL (*p* < 0.001), PSA ≥ 100 ng/mL prior to cabazitaxel treatment (*p* = 0.002) were significantly associated with shorter OS (Table 3). There was no significant correlation of patient age (*p* = 0.537, Fig. 1B), the response to prior docetaxel therapy (*p* = 0.339, Fig. 1C), cycles of docetaxel therapy (*p* = 0.379, Fig. 1D), or EOD score (*p* = 0.120, Supplementary Fig. 1) with shorter OS.Multivariate analysis revealed that, poor PS (HR = 5.667; CI 1.81–17.74, *p* = 0.003), and visceral metastasis (HR = 2.939; CI 1.173–7.367, *p* = 0.021) were independent prognostic indicators for OS.

**Table 3:** Results of univariate and multivariate analysis influencing OS

Variables	*n* (%)	Univariate analysis	Multivariate analysis		
		*p* value	HR	95% CI	*p* value
Age					
≤ 75	39 (83.0)	0.537			
75-years	8 (17.0)				
PS					
1, 2	7 (14.9)	< 0.001	5.667	1.81–17.74	0.003
0	40 (85.1)				
Visceral metastasis					
Yes	13 (27.7)	0.129	2.939	1.173–7.367	0.021
No	34 (72.3)				
PSA (ng/mL)					
≧ 100	24 (51.1)	0.002			
< 100	23 (48.9)				
Hb (mg/dL)					
≤ 11	19 (40.4)	< 0.001			
> 11	28 (59.6)				
ALP (IU/L)					
> 350	19 (40.4)	0.268			
≤ 350	28 (59.6)				
EOD score					
≧ 2	26 (55.3)	0.120			
< 2	21 (44.7)				
Cycles of docetaxel therapy					
≧ 10	17 (36.2)	0.379			
< 10	30 (63.8)				
Response to docetaxel therapy					
Yes	28 (59.6)	0.339			
No	19 (40.4)				

## Discussion

Cabazitaxel was the first agent demonstrating a survival benefit in Western CRPC patients progressing during or after docetaxel [1, 8, 9]. A phase I cabazitaxel study in Japan demonstrated the safety and the efficacy of PSA-PFS [8, 9]. Cabazitaxel has been widely applied for the treatment of mCRPC patients in Japan as well [6, 7], although that study in Japan did not demonstrate the efficacy and the prognostic indicators in relation to OS. Moreover, the tolerability of Asian patients to chemotherapeutic agents was reported to be generally worse than that of Western patients [1, 9, 10]. Therefore, we conducted a retrospective assessment of oncological outcomes in 47 patients with mCRPC who received cabazitaxel therapy after the failure of a docetaxel-containing regimen, focusing on patient’s age. In clinical trial, eligibility criteria usually exclude elder patients or higher comorbidities. Thus, it is difficult to translate trial treatment outcomes in a real-world setting. It should be acknowledged that the need for age-based exclusion has been called into question [11, 12]. Following treatment with cabazitaxel therapy, 20 (42.6%) were judged to have responded to cabazitaxel with a PSA decrease ≥ 30% from the baseline. A 30% PSA response to cabazitaxel was achieved in 4 (50.0%) patients with  ≧ 75 years (*n* = 8) and 16 (41.0%) patients with less than 75 years (*n* = 39). Considering the introduction of cabazitaxel in elder patients outside of clinical trials, the International Society of Geriatric Oncology shows appropriate recommendation which says elder patients need to be managed according to individual health status rather than chronological age (Fig. 1B) [13, 14]. Our results indicate that it should be acknowledged that age alone should not prevent patients deriving benefit from cabazitaxel therapy.

The oncological outcomes achieved in our series were as follows: PSA decline of > 30%, 20 (42.6%); median OS, 16.1 months. These findings are comparable to those of the TROPIC study (PSA response rate, 39.2%; median OS, 15.1 months) or studies retrospectively conducted in Western counties [1, 5, 15]. PSA decline of > 30% was observed in 20 (42.6%), respectively. These findings indicate that cabazitaxel therapy to Japanese mCRPC patients progressing after docetaxel could result in oncological outcomes comparable to those of Western mCRPC patients. Although Cabazitaxel showed activity in both docetaxel-sensitive and docetaxel-resistant cancers in preclinical testing and in clinical trials was clearly demonstrated in the TROPIC trial [1–3, 16], the association of efficacies between docetaxel and cabazitaxel in Japanese CRPC patients has been unclear. To assess the association of efficacies between docetaxel and cabazitaxel in this study thought to be informative for the patient to decision the introduction of Cabazitaxel. In this series, for the first time, we demonstrated that there was no significant correlation of PSA response rate of pre-treated docetaxel between docetaxel and cabazitaxel. In addition, treatment cycles of pre-treated docetaxel may not be indicative of OS in cabazitaxel. These results suggest that it need not consider the sensitivity of docetaxel when determining agents following the failure of docetaxel. Among the several factors examined in this study, ECOG PS and visceral mets were identified as one of the independent predictors of OS after the introduction of cabazitaxel. These results suggest the importance of earlier introduction of cabazitaxel irrespective of PSA response during docetaxel therapy (Table 3).

We would like to describe several limitations in our study. The study design was retrospective and involved a relatively small population of Japanese mCRPC patients. Therefore, the findings obtained in this study should be verified in a prospective study including other ethic groups based on our study. Secondly, despite the use of cabazitaxel in all of the included patients with docetaxel-refractory disease, several types of sequential therapy were applied to these patients in this study, which may affect the present outcomes.

## Conclusion

These results indicate that the introduction of cabazitaxel for Japanese mCRPC patients could result in oncological outcomes equivalent to those in Western populations without any association with patient’s age and the profiles of previous docetaxel therapy.

## Electronic supplementary material

Below is the link to the electronic supplementary material.

**Supplementary Fig 1**. D: Kaplan–Meier for time-to-overall survival in the EOD score specified population (n=47) (JPG 37 KB)

## References

[1] de Bono JS, Oudard S, Ozguroglu M (2010). Prednisone plus cabazitaxel or mitoxantrone for metastatic castration-resistant prostate cancer progressing after docetaxel treatment: a randomised open-label trial. Lancet.

[2] Mita AC, Denis LJ, Rowinsky EK (2009). Phase I and pharmacokinetic study of XRP6258 (RPR 116258A), a novel taxane, administered as a 1-hour infusion every 3 weeks in patients with advanced solid tumors. Clin Cancer Res.

[3] Pivot X, Koralewski P, Hidalgo JL (2008). A multicenter phase II study of XRP6258 administered as a 1-h i.v. infusion every 3 weeks in taxane-resistant metastatic breast cancer patients. Ann Oncol.

[4] Pezaro CJ, Omlin AG, Altavilla A (2014). Activity of cabazitaxel in castration-resistant prostate cancer progressing after docetaxel and next-generation endocrine agents. Eur Urol.

[5] Al Nakouzi N, Le Moulec S, Albiges L (2015). Cabazitaxel remains active in patients progressing after docetaxel followed by novel androgen receptor pathway targeted therapies. Eur Urol.

[6] Kosaka T, Oya M (2015). Hemorrhagic cystitis in a patient without a past history of radiation therapy who was treated with cabazitaxel for CRPC. Ann Oncol.

[7] Watanabe K, Kosaka T, Hongo H, Tamaki S, Oya M (2017) Headache caused by brain metastases of castration-resistant prostate cancer during cabazitaxel therapy. Keio J Med 10.2302/kjm.2016-0014-CR 28392539

[8] Mukai H, Takahashi S, Nozawa M (2014). Phase I dose-escalation and pharmacokinetic study (TED 11576) of cabazitaxel in Japanese patients with castration-resistant prostate cancer. Cancer Chemother Pharmacol.

[9] Nozawa M, Mukai H, Takahashi S (2015). Japanese phase I study of cabazitaxel in metastatic castration-resistant prostate cancer. Int J Clin Oncol.

[10] Shigeta K, Kosaka T, Yazawa S (2015). Predictive factors for severe and febrile neutropenia during docetaxel chemotherapy for castration-resistant prostate cancer. Int J Clin Oncol.

[11] Heidenreich A, Bracarda S, Mason M (2014). Safety of cabazitaxel in senior adults with metastatic castration-resistant prostate cancer: results of the European compassionate-use programme. Eur J Cancer.

[12] Mukherji D, Pezaro CJ, Shamseddine A, De Bono JS (2013). New treatment developments applied to elderly patients with advanced prostate cancer. Cancer Treat Rev.

[13] Droz JP, Aapro M, Balducci L (2014). Management of prostate cancer in older patients: updated recommendations of a working group of the International Society of Geriatric Oncology. Lancet Oncol.

[14] Droz JP, Balducci L, Bolla M (2010). Management of prostate cancer in older men: recommendations of a working group of the International Society of Geriatric Oncology. BJU Int.

[15] Heidenreich A, Scholz HJ, Rogenhofer S (2013). Cabazitaxel plus prednisone for metastatic castration-resistant prostate cancer progressing after docetaxel: results from the German compassionate-use programme. Eur Urol.

[16] Kosaka T, Hongo H, Miyazaki Y, Nishimoto K, Miyajima A, Oya M (2017). Reactive oxygen species induction by cabazitaxel through inhibiting Sestrin-3 in castration resistant prostate cancer. Oncotarget.

