# Supplementary figures and images for: No significant impact of patient age and prior treatment profile with docetaxel on the efficacy of cabazitaxel in patient with castration-resistant prostate cancer
Source: Cancer Chemother Pharmacol. 2018 Oct 3;82(6):1061–6. doi: 10.1007/s00280-018-3698-1 (PMC6267665; doi:10.1007/s00280-018-3698-1)

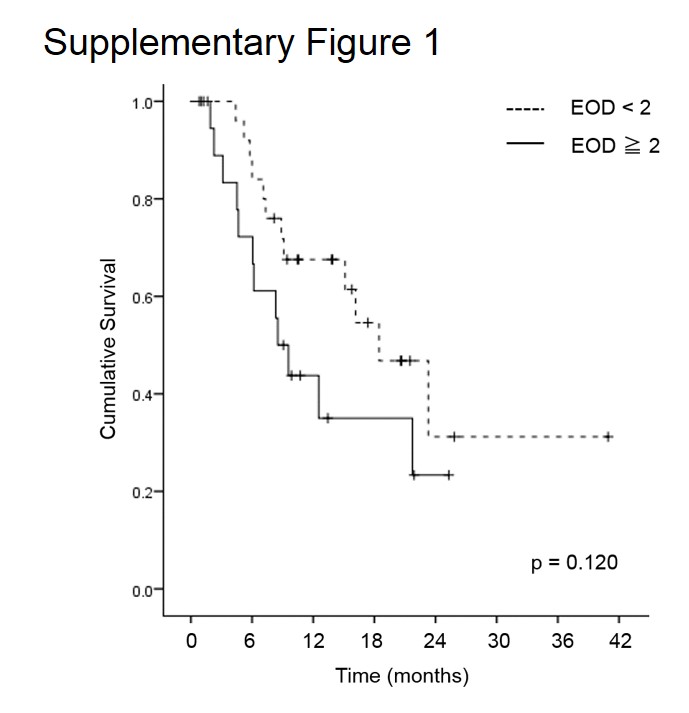

Supplement: Supplementary file 1 — Supplementary Fig 1. D: Kaplan–Meier for time-to-overall survival in the EOD score specified population (n=47) (JPG 37 KB) [file 280_2018_3698_MOESM1_ESM.jpg]
